# Disentangling the Neuroanatomical Correlates of Perseveration from Unilateral Spatial Neglect

**DOI:** 10.3233/BEN-2012-110235

**Published:** 2013

**Authors:** Jonathan T. Kleinman, Jeffery C. DuBois, Melissa Newhart, Argye E. Hillis

**Affiliations:** ^1^Departments of NeurologyJohns Hopkins University School of MedicineBaltimoreMDUSA; ^2^Physical Medicine and RehabilitationJohns Hopkins University School of MedicineBaltimoreMDUSA; ^3^Department of Cognitive ScienceJohns Hopkins University School of MedicineBaltimoreMDUSA; ^4^Stanford University School of MedicineStanfordCAUSA

**Keywords:** Perseveration, neglect, acute stroke, diffusion-weighted imaging, perfusion-weighted imaging

## Abstract

Perseverative behavior, manifest as re-cancelling or re-visiting targets, is distinct from spatial neglect. Perseveration is thought to reflect frontal or parietal lobe dysfunction, but the neuroanatomical correlates remain poorly defined and the interplay between neglect and perseveration is incompletely understood. We enrolled 87 consecutive patients with diffusion-weighted, perfusion-weighted imaging, and spatial neglect testing within 24 hours of right hemisphere ischemic stroke. The degrees of spatial neglect and perseveration were analyzed. Perseveration was apparent in 46% (40/87) of the patients; 28% (24/87) showed perseveration only; 18% (16/87) showed both perseveration and neglect; and 3% (3/87) showed neglect only. Perseverative behaviors occur in an inverted “U” shape: little neglect was associated with few perseverations; moderate neglect with high perseverations; and in severe neglect targets may not enter consciousness and perseverative responses decrease. Brodmann areas of dysfunction, and the caudate and putament, were assessed and volumetrically measured. In this study, the caudate and putamen were not associated with perseveration. After controlling for neglect, and volume of dysfunctional tissue, only Brodmann area 46 was associated with perseveration. Our results further support the notion that perseveration and neglect are distinct entities; while they often co-occur, acute dorsolateral prefrontal cortex ischemia is associated with perseveration specifically.

## 1. Introduction

Perseveration is an experience, activity, continuation or recurrence of a behavior that inappropriately persists in the absence of external stimuli or after task demands change [1–4]. It can manifest as repetitive behaviors, fixations, revisitings, cancelling of targets, or elaboration on drawings in the ipsilesional hemispace [4,5]. While in general neglect is a defective or "negative" manifestation, perseveration is a productive or "positive" manifestation of spatial bias [6]. Perseveration often co-occurs with unilateral spatial neglect, which is often defined as the inability to attend or respond to space contralateral to brain damage, not attributable to a primary sensory or motor deficit [7]. Despite these concrete descriptions, both perseveration and neglect are heterogeneous disorders that can occur due to the breakdown of many cognitive processes. As a result, processing breakdowns manifest themselves distinctly in each test of neglect and also have distinct underlying neuroanatomical correlates that are described elsewhere [9–11]. Additionally, each deficit may serve to exacerbate other processing dysfunctions. Posner and colleagues (1984) hypothesized that neglect consists of a bias in in spatial attention toward the ipsilesional side and a failure to disengage from the ipsilesional side of space [12]. Another hypothesis is that both perseveration and neglect appear independently [13]. Sandson and Albert (1987) proposed that perseverative behav-

ISSN 0953-4180/13/$27.50 © 2013 – IOS Press and the authors. All rights reserved iors fit into three categories: (1) recurrent perseverations, (2) stuck-in-set perseveration (inability to shift categories to perform a task), (3) continuation of a current behavior [4]. Manly and colleagues (2002) hypothesized that patients with neglect show perseveration due to implicit knowledge of contralesional stimuli [14]. Despite being reported in 30–90% of patients with right hemisphere stroke, perseveration has received less attention than neglect [6,15–17]. Clinically, perseveration interferes with functional tasks, rehabilitation, and may indicate a failure to shift attention [6,15,17,18].

There are different ways to detect and study perseveration. Researchers have studied perseverations defined as "re-markings" or crossing out stimuli many times [6, 14,15,17,19,20]. These "re-markings" have been defined as "simple" often re-cancelling of previous targets, or "complex" such as elaborate drawings [6,15, 21]. It is also possible to examine "re-visting" of previously visited targets using computerized tasks when no physical mark is left, which may be more dependent on spatial working memory [16,22]. Many patients studied using computerized "re-visiting" tasks did not have unilateral spatial neglect as assessed with paper and pencil tasks, indicating that if they had spatial bias, it was not captured on these tasks. It should not be assumed that this dysfunctional "re-visiting" behavior is the same as that seen in "re-marking" tasks [14].

Perseveration and neglect often co-occur; and recently, the interaction between neglect and perseveration has been appreciated. Perseveration can occur in patients without *neglect* on paper and pencil tasks [17, 20], although it may still be caused by spatial bias [6]. The severity of neglect is thought to correlate with the degree of perseverations [14–16,18,19]. In an elegant experiment, Manly and colleagues (2002) removed left sided stimuli and showed that as the number of contralesional targets was decreased, both neglect and perseveration decreased [14]. Surprisingly, this study also showed that only removal of target stimuli decreased perseveration, indicating that some high-level stimuli information is processed. Although perseverations are not affected by the number of contralesional stimuli in all patients [6,18,20,21]. Ipsilesional monocular patching has been shown to decrease the number of neglected stimuli, but has no effect on the number of perseverations [23]. Prism adaptation surprisingly has been shown to decrease severity of neglect, increase the severity of perseveration, and shift perseverations contralesionally in one patient [24], although perseveration is not increased uniformly in all patients while using prism glasses [21].

Given the intricate and complicated relationship between neglect and perseveration, it is important to consider the neuroanatomical correlates of both when attempting to study perseveration. In comparison to unilateral spatial neglect, the neuroanatomical correlates of perseveration remain poorly defined and may depend on how perseveration is assessed (i.e. task design). In "re-visiting" tasks, Mannan and colleagues (2005) showed that the intraparietal sulcus was associated with high "re-visiting" rates as time increased, possibly due to working memory deficits in patients with high rates of re-visiting [16]. "Re-visiting" tasks may be more dependent on spatial working memory function than some other tasks, but patients with frontal lesions showed pathologic "re-visiting" rates irrespective of time [16]. In re-marking tasks, anterior and frontal lesions have been shown to be associated with perseveration [6,15] although after controlling for volume of lesion this association was not found by others [17]. Others have found that damage to the basal ganglia was associated with persevertaion in subacute stroke patients [6,17]. In both cases, the locations and not absolute volume of damage was most critical [16]. These studies have evaluated patients in the subacute or chronic stage of stroke, after patients may have shown some degree of reorganization of structure/function relationships.

Therefore, we sought to disentangle unilateral spatial neglect from perseveration in right hemisphere ischemic stroke, and to define the neuroanatomical correlates of acute perseveration on a cancellation task in the first 24 hours of stroke onset, before the opportunity for reorganization or recovery. We addressed this aim by identifying areas of tissue dysfunction on diffusion-weighted and perfusion-weighted MRI significantly associated with perseveration and neglect (P+N+), perseveration without neglect (P+N-), and neither perseveration or neglect (P-N-) in a consecutive series of patients with acute ischemic right hemisphere stroke.

## 2. Methods

### 2.1. Population

Consecutive right hemisphere stroke patients with diffusion weighted imaging (DWI), perfusion weighted imaging (PWI), and spatial neglect testing within 24 hours of symptom onset were included in this study. All patients provided informed consent for the study, using methods and consent forms approved by the Johns Hopkins Institutional Review Board. Pa-

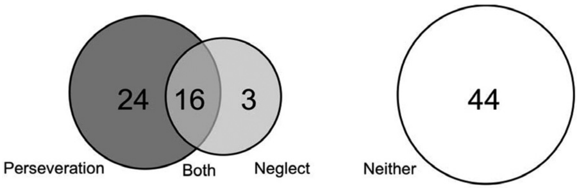

Fig. 1. The frequency and overlap of neglect and perseveration.

tients were excluded if they had any contraindication to MRI, prior dementia, other neurological disease, reduced level of consciousness, sedation, and any left sided or infratentorial stroke or hemorrhage on MRI.

### 2.2. Cognitive tasks

A modified line cancellation was administered 25 cm from the patient at center and 45 degrees right and left of center [25]. Patients were asked to cross out each line *only* one time. Neglect was defined as omission of $> 10\%$ lines on the line cancellation task, with majority of omission on the left, as no normal subject made $> 10\%$ omissions [26]. Perseveration was defined as $1+$ re-marking on the line cancellation task, as defined in previous reports [15,17]. A test administrator ensured that responses in which the line was crossed twice during the same pen-stroke were not considered perseverations.

### 2.3. Image analysis

Imaging acquisition information is presented elsewhere [27]. PWI scans were post-processed to generate maps of time to peak (TTP) arrival of the GdDTPA in each voxel. Tissue dysfunction was defined as dense ischemia (bright in DWI, dark on ADC maps) and/or hypoperfusion (defined as $\geqslant 4$ s mean delay in TTP arrival of contrast across voxels in the region of interest relative to the homologous region in the contralateral hemisphere) based on evidence that this tissue is dysfunctional, although it may not be at risk for infarction [26–28]. Diffusion and perfusion scans were analyzed to determine both total volume of tissue dysfunction, measured with Image J (http://rsb.info.nih.gov/ij), and presence or absence of tissue dysfunction in 17 right hemisphere Brodmann areas (BA) defined by a published atlas [29], and in the caudate, putamen, and insula. The dysfunctional volume used for analysis was the larger of either the diffusion or perfusion measure.

### 2.4. Statistical analysis

Chi-square tests were used to identify which BA of dysfunction were significantly different between groups. For continuous variables two-tailed t-tests were used. A p-value $< 0.05$ was considered statistically significant. We did not adjust for multiple testing as this was an exploratory analysis. Linear regression was performed to determine if the number of perseverations correlated with the presence or absence of dysfunction in areas predicted previously. Logistic regression model was then used to evaluate the associations between perseveration with and without neglect (dependent variable) with BA (independent variable) identified using chi-square tests. Models included adjustment for age and volume. Analysis of variance (ANOVA) was used to compare the volume of abnormalities on DWI and PWI between the three groups ((P+N+, P+N-, and P-N-). Repeated measures ANOVA was used to check the effects of hemispace on the number of perseverative responses. ANOVA models were used to evaluate the association between the number of perseverations (dependent variable) with Brodmann areas identified previously. We also evaluated whether number of perseverations were associated with area of space that the task was presented (center, left, or right) and group (P+N+ or P+N-) using repeated measures ANOVA.

## 3. Results

### 3.1. Population

We enrolled 87 patients: 53% of the patients were men, average age 60.8 $\pm$ 15.2 years (range: 23–85); and average education was 12$^{\text{th}}$ grade (range: 3–20 years). Forty patients (46%) showed perseveration (P+): 24 perseverated only (P+N-); 16 showed both perseveration and neglect (P+N+). Only three patients showed neglect without perseveration (P-N+) (Fig. 1). There was no gender difference in the presence or frequency of perseveration. Examples of perseveration without neglect and perseveration with neglect are depicted in Fig. 2. Patients with perseveration tended to be older than those without perseveration, although not significantly (64 vs 58 $p = 0.07$), but patients with neglect were not older than those without (64 vs 59.9, $p = 0.34$). The average volume of dysfunctional tissue (infarct and/or hypoperfusion) in the population was 29.8 $\pm$ 51.1 cc (Table 1). For those with perseveration the average was 37.9 $\pm$ 54.0 cc versus 19.5 $\pm$ 39.3 cc for those without perseveration ($p = 0.049$). For those with neglect the volume was 70.0 $\pm$ 70.6 cc versus 18.4 $\pm$ 37.7 cc for without neglect ($p = 0.006$). Gender was not associated with perseveration or neglect.

Table 1
Areas associated with neglect and perseveration. Brodmann areas associated with perseveration and neglect using univariate analysis. The strongest associations with brain regions were found for neglect, and may be "driving" the observed associations found in the perseveration group

| Brodmann's Areas | P- $n = 47(\%)$ | P+ $n = 40(\%)$ | **p value** | N- $n = 68(\%)$ | N+ $n = 19(\%)$ | **p value** | P+N- $n = 24(\%)$ | P+N+ $n = 16(\%)$ | **p value** |
|---|---|---|---|---|---|---|---|---|---|
| BA 4 | 7 (14.9) | 12 (30.0) | **0.09** | 10 (14.7) | 9 (47.4) | **0.005** | 4 (16.7) | 8 (50.0) | **0.034*** |
| BA 6 | 13 (27.7) | 16 (40.0) | **0.22** | 18 (26.5) | 11 (57.9) | **0.01** | 6 (25.0) | 10 (62.5) | **0.018** |
| BA 7 | 4 (8.5) | 6 (15.0) | **0.50*** | 7 (10.3) | 3 (15.8) | **0.68*** | 3 (12.5) | 3 (18.8) | **0.67*** |
| BA 10 | 1 (2.1) | 2 (5.0) | **0.59** | 1 (1.5) | 2 (10.5) | **0.12*** | 0 | 2 (12.5) | **0.15*** |
| BA 11 | 0 | 2 (5.0) | **0.46*** | 0 | 1 (5.3) | **0.22*** | 0 | 1 (6.3) | **0.40*** |
| BA 18 | 8 (17.0) | 9 (22.5) | **0.52** | 9 (13.2) | 8 (42.1) | **0.009*** | 2 (8.3) | 7 (43.8) | **0.0175*** |
| BA 19 | 10 (21.3) | 15 (37.5) | **0.096** | 15 (22.1) | 10 (58.8) | **0.009** | 6 (25.0) | 9 (56.3) | **0.0455** |
| BA 20 | 1 (2.1) | 3 (7.5) | **0.33** | 2 (2.9) | 2 (10.5) | **0.21*** | 1 (4.2) | 2 (12.5) | **0.55*** |
| BA 21 | 6 (12.8) | 9 (22.5) | **0.23** | 8 (11.8) | 7 (36.8) | **0.017*** | 3 (12.5) | 6 (37.5) | **0.12*** |
| BA 22 | 12 (25.5) | 19 (47.5) | **0.03** | 20 (29.4) | 11 (57.9) | **0.02** | 9 (37.5) | 10 (62.5) | **0.12** |
| BA 37 | 11 (23.4) | 18 (45.0) | **0.03** | 15 (22.1) | 14 (73.7) | $<$ **0.0001** | 5 (20.8) | 13 (81.3) | **0.0002** |
| BA 38 | 3 (6.4) | 5 (12.5) | **0.46*** | 4 (5.9) | 4 (21.1) | **0.065*** | 1 (4.2) | 4 (25.0) | **0.14*** |
| BA 39 | 9 (19.2) | 16 (40.0) | **0.03** | 16 (23.5) | 9 (47.4) | **0.04** | 8 (33.3) | 8 (50.0) | **0.29** |
| BA 40 | 8 (17.0) | 18 (45.0) | **0.0045** | 15 (22.1) | 11 (57.9) | **0.003** | 8 (33.3) | 10 (62.5) | **0.069** |
| BA 44 | 4 (8.5) | 10 (25.0) | **0.037** | 6 (8.8) | 8 (42.1) | **0.002*** | 2 (8.3) | 8 (50.0) | **0.0068*** |
| BA 45 | 4 (8.5) | 8 (20.0) | **0.12** | 5 (7.4) | 7 (36.8) | **0.003*** | 2 (8.3) | 6 (37.5) | **0.042*** |
| BA 46 | 2 (4.3) | 9 (22.5) | **0.01** | 4 (5.9) | 7 (36.8) | **0.0016*** | 2 (8.3) | 7 (43.8) | **0.0175*** |
| Caudate | 11 (23.4) | 8 (20.0) | **0.80** | 14 (20.6) | 5 (26.3) | **0.75** | 15 (62.5) | 4 (25.0) | **0.74** |
| Putamen | 11 (23.4) | 8 (20.0) | **0.80** | 14 (20.6) | 5 (26.3) | **0.75** | 15 (62.5) | 4 (25.0) | **0.74** |
| Caudate and/or Putamen | 14 (29.8) | 12 (32.5) | **1.00** | 20 (29.4) | 6 (31.6) | **1.00** | 21 (87.5) | 5 (31.3) | **1.00** |
| insula | 10 (21.3) | 17 (42.5) | **0.03** | 17 (25.0) | 10 (52.6) | **0.02** | 8 (33.3) | 9 (56.3) | **0.15** |
| anterior insula | 8 (17.0) | 12 (30.0) | **0.15** | 11 (16.2) | 9 (47.4) | **0.01*** | 4 (16.7) | 8 (50.0) | **0.0367*** |
| Dysfunctional volume | $19.5 \pm 39.3$ cc | $37.8 \pm 54.0$ cc | **0.049**** | $18.4 \pm 37.7$ cc | $70.0 \pm 70.6$ cc | **0.006**** | $21.0 \pm 45.5$ cc | $73.11 \pm 69.8$ cc | **0.014**** |

*Based on Fisher's exact test as expected cell frequencies are $< 5$ in some cells. **Based on ttest.

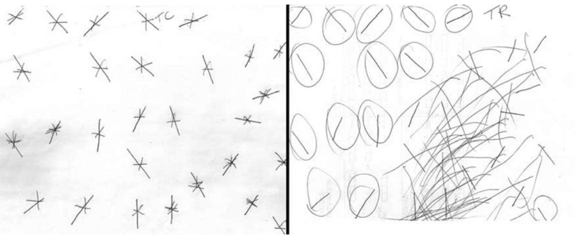

Fig. 2. Perseveration without neglect (left), and perseveration with spatial neglect (right). Circled lines were those that were omitted (uncrossed) by the patient.

### 3.2. Perseveration

The highest mean number of perseverations occurred during presentation in the center space versus the left or right space although this was not statistically significant (F(2,36) $= 1.16$, $p = 0.324$). Similarly, within subjects, the location of presentation did not affect the number of perseverations (F(2,35) $= 1.36$, $p = 0.27$). The location of perseverations on the presented page was primarily within the right (ispsilesional hemispace) (Fig. 3). One-way ANOVA showed the number of perseverations in the three locations differed from each other ($p < 0.001$). Both the left side and center of the page differed from the right side in terms of percent of total within-subject perseverations (19.2 & 18.5% versus 62.3%, $p < 0.001$ for both). The left side of the page did not differ from the center of the page in terms of percent of perseveration (19.2 vs 18.5% of perseverations, $p = 0.89$).

### 3.3. Perseveration and neglect

Some patients showed perseverative behavior without neglect documented on paper and pencil tasks. P+N- subjects showed fewer perseverations than P+N+ subjects ($p = 0.007$) (Fig. 4). Within subjects, the rate of neglect errors influenced the number of perseverations (F(1,36) $= 8.64$, $p = 0.006$). Additionally, the severity of neglect showed an inverted "U" shaped relationship with perseveration. Little neglect was associated with few perseverations, intermediate levels of neglect were associated with the highest number of perseverations, and extreme neglect was associated with again fewer perseverations (Fig. 5). The influence of neglect on persevaration did not depend on the location of presentation (F(2,35) $= 0.43$, $p = 0.65$).

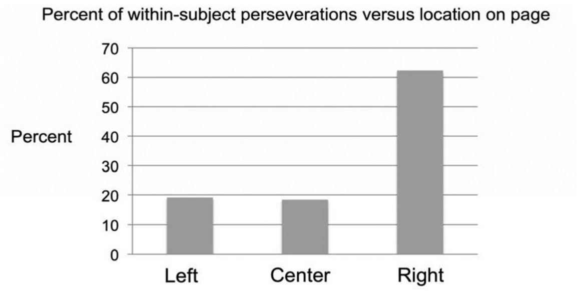

Fig. 3. Within-subject percent of total perseverations based upon location on the page of presentation.

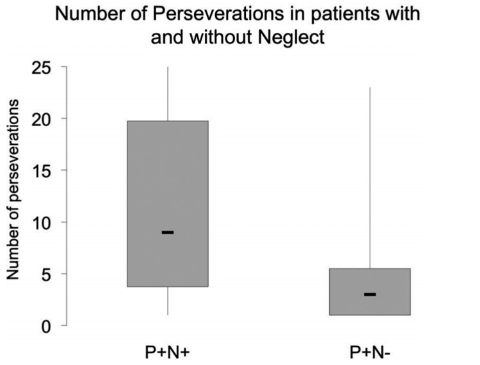

Fig. 4. Number of perseverations in P+N+ and P+N- subjects.

### 3.4. *Neuroanatomical correlates of perseverative behaviors*

Without taking into account neglect or correcting for volume of dysfunctional tissue, the areas of the brain most highly associated with perseveration are presented in Table 1. The areas associated with neglect are also presented in Table 1. Dysfunctional tissue was significantly larger in P+N+ versus P+N- groups ($p = 0.014$). The strongest correlations were found for neglect, therefore when comparing P+N+ to P+N- we see the locations that are more associated with neglect than perseveration. These BA are: 4, 6, 18, 19, 37, 44, 45, 46, and the anterior insula. The converse contrast (P+N+ compared to P-N+) did not reveal areas significantly more associated with perseveration than neglect because only three patients had P-N+. Dysfunction in the caudate, was not associated with perseveration, neglect, or P+N+. Dysfunction in the putamen was also not associated with perseveration, neglect, or P+N+. Dysfunction in the putamen and/or caudate was not associated with any behaviors assessed.

Using linear regression, after correction for age and dysfunctional tissue volume, only dysfunction in BA

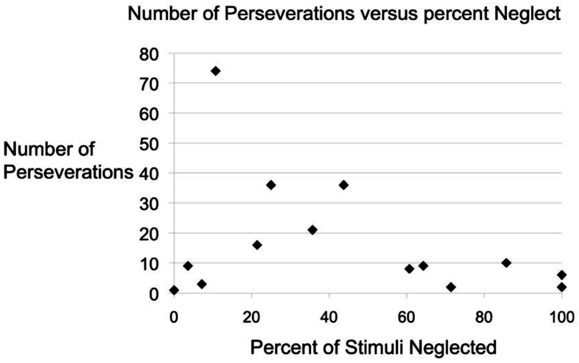

Fig. 5. Neglect percent versus the total number of perseverations.

46 was associated with perseveration ($\beta = 5.23$, $p = 0.023$). The mean difference in number of perseverations between patients with dysfunction in BA46 versus no dysfunction is 5.2 perseverations.

## 4. Discussion

We examined perseveration at the earliest time point following damage yet studied, before substantial reorganization of structure/function relationships or rehabilitation could take effect. Other strengths of this dataset are a relatively large number of patients, with both diffusion and perfusion scans, dysfunctional tissue analysis, and assessment of both perseveration and neglect. Many recent studies have been performed in the chronic phase [4,16,19,20,23,24] or subacute phase [6, 14,17] after some patients may have recovered from perseverative behavior, making it difficult to ascertain whether or not the patients' lesions were critical for the early appearance of perseveration. When studying in the acute or early subacute phase, both diffusion and perfusion must be used to account for all dysfunctional tissue that might account for the abnormal behavior. Additionally, we examined patients both with and without neglect, with and without perseveration, and the combinations of those possibilities as shown in Fig. 1 in an attempt to disentangle this interaction.

In this population, there was no statistically significant age difference between patients with and without perseveration, although the average age of patients with perseveration was higher, similar to what is seen in neglect [30]. Similar to recent neglect studies, there was no statistical difference between and gender and proportion of perseveration [31,32] although gender differences have been reported in neglect in some studies [17].

Perseveration was higher in P+N+ patients versus P+N- patients similar to previous studies. When comparing studies of perseveration it is important to recognize the severity of neglect as a possible cause for discordant results. In a study by Mannan and colleagues (2005) only one patient out of 16 had paper and pencil perseverative behavior [16]. In contrast to previous studies, we show the relationship is not linear. We found a unique pattern of perseverative behavior with the highest degree of perseveration in patients with mild to moderate neglect. Thus the interaction between neglect and perseveration forms an inverted "U". Although not statistically different, upon close examination, Rusconi and colleagues (2002) show the same highest perseveration in the ipsilesional middle column of four [6]. Deficits in visual fields cannot account for these results as it has long been known that hemianopsia does not produce neglect, and does not seem to produce perseverations [6,33].

Similar to previous studies we show that the location of perseverations is mainly restricted to the ipsilesional hemispace (Fig. 3). Our results are consistent with those of Rusconi and colleagues (2002), who showed perseveration in subjects without neglect, signifying persistent spatial bias despite adequate performance on tasks of neglect [6]. In subjects with neglect, attention is not distributed normally until the midline, but at varying rates decays toward the contralesional hemispace [34–36]. Using prism glasses in a patient with severe neglect, Nys and colleagues (2008) showed that perseveration location shifted towards the contralesional side [24]. Therefore, the most significant neglect was associated with only ipsilesional perseveration, but through prism adaptation perseveration occurred in both ipsilesional and contralesional hemispace. Although this assocation was not found by others [21], it may be due to the severity of neglect each patient had prior to treatment. Therefore, both the results of monocular patching and prism adaptation may be explained by modifying the percent of stimuli that are reaching both attention and full conscious awareness. This concept is not explained by simply the number of stimuli on the left side of the page as shown by Manley and colleagues (2002) [14]. They varied the number of irrelevant "stimuli" and "targets" on the contralesional (left) side of the page, and showed that it is only the number of "targets" that modify the extent of perseverative behaviors.

These data also shed new light on the localization of perseveration. Perseveration was predicted only by tissue dysfunction in right BA 46 using linear regression. That is, in our population, ischemia in right BA 46 was associated increased perseverative responses. Therefore, although perseveration occurred in patients without lesions to BA 46, in patients with ischemia in this region the perseverative responses were more common. In contrast to previous studies, the caudate and putamen were not associated with either neglect or perseveration, nor any combination of the two [17]. It is possible that in the subacute phase there is an association (indicating that these structures may be critical to recovery from perseveration), even when the association is not present in the acute phase. Likewise, subcortical infarction does not seem to account for unilateral spatial neglect in acute stroke, but often the cortical areas of hypoperfusion that co-occurs with subcortical infarcts can account for the neglect [8]. Nys and colleagues (2006) were not able to evaluate the relative importance of specific regions within the frontal lobe, but suggested dorsolateral prefrontal cortex (BA 46) was most likely implicated due to its involvement in non-motor perseveration [17]. Mannan and colleagues (2005) showed that lesions involving the inferior frontal and intraparietal sulcus were associated with revisiting targets, a type of perseveration that might influenced by impaired working memory [16]. We found that only specific regions within posterior frontal cortex (including dorsolateral prefrontal cortex) were associated with perseveration involving repetitive cancelation of targets. This study was conducted only in right hemisphere stroke, but in previous studies no difference was found in the prevalence of perseveration after either right or left hemisphere damage, whether or not neglect was present.

Limitations of our study include an inability to identify distinct types of perseveration, and no evaluation of left hemisphere strokes, although right spatial neglect occurs in a significant proportion of patients acutely following left hemisphere stroke [9,31]. Nys and colleagues (2006) found that perseveration occurred at similar rates following damage to each hemisphere, although right neglect increased central perseverations and left neglect increased right-sided perseverations [17]. Not all investigators have found high perseveration rates following left hemisphere damage [6]. Second, we did not calculate an index of neglect severity as in some recent studies [37,38]. Although we feel the absolute number of neglect responses is an appropriate measure and the data is clear (Fig. 5). The accurate assessment of multiple subtypes of "neglect" and erros on distinct tasks was not the focus of this paper, although the assessment of neglect presented is limited [8,10,11]. Additionally we used a relatively loose description of perseveration (1+ remarkings) to allow comparison between our results and previous studies of perseveration. In an attempt to go beyond this binary assessment of perseveration, we correlated the severity of perseveration with dysfunction in BA 46.

In summary, the highest degree of perseveration was found with moderate levels of spatial neglect, and the relationship between neglect and perseveration formed an inverted "U" shape. We found no gender difference, and a trend toward increased age in patients with both perseveration and neglect. Even without controlling for other variables, the caudate and putamen were not associated with either perseveration or neglect. Dysfunction in BA 46 was the only ischemic area associated with perseveration, but this only explained a small portion of perseverative behavior. Future studies are needed to confirm the neuroanatomical correlates of perseveration and to identify effective treatment strategies.

## Acknowledgments

We thank Rita Popat for help with statistical analysis for this article. We are grateful to the patients who participated in this study.

## Funding

This work was supported by: The Stanford School of Medicine Medical Scholars Fund [JTK]; The Dean's Scholarship for Student Research [JCD]; and the National Institute of Neurological Disorders and Stroke [RO1NS47691 to AEH].

## Conflict of interest

The authors have no conflicts of interest, relationships, affiliations or other support relevant to subject of the manuscript to disclose.
